# Feasible Introgression of an Anti-pathogen Transgene into an Urban Mosquito Population without Using Gene-Drive

**DOI:** 10.1371/journal.pntd.0002827

**Published:** 2014-07-03

**Authors:** Kenichi W. Okamoto, Michael A. Robert, Fred Gould, Alun L. Lloyd

**Affiliations:** 1 Department of Entomology, North Carolina State University, Raleigh, North Carolina, United States of America; 2 Department of Mathematics and Biomathematics Graduate Program, North Carolina State University, Raleigh, North Carolina, United States of America; 3 Department of Biology and the Department of Mathematics and Statistics, University of New Mexico, Albuquerque, New Mexico, United States of America; 4 Fogarty International Center, National Institutes of Health, Bethesda, Maryland, United States of America; The Pennsylvania State University, United States of America

## Abstract

**Background:**

Introgressing anti-pathogen constructs into wild vector populations could reduce disease transmission. It is generally assumed that such introgression would require linking an anti-pathogen gene with a selfish genetic element or similar technologies. Yet none of the proposed transgenic anti-pathogen gene-drive mechanisms are likely to be implemented as public health measures in the near future. Thus, much attention now focuses instead on transgenic strategies aimed at mosquito population suppression, an approach generally perceived to be practical. By contrast, aiming to replace vector competent mosquito populations with vector incompetent populations by releasing mosquitoes carrying a single anti-pathogen gene without a gene-drive mechanism is widely considered impractical.

**Methodology/Principal Findings:**

Here we use Skeeter Buster, a previously published stochastic, spatially explicit model of *Aedes aegypti* to investigate whether a number of approaches for releasing mosquitoes with only an anti-pathogen construct would be efficient and effective in the tropical city of Iquitos, Peru. To assess the performance of such releases using realistic release numbers, we compare the transient and long-term effects of this strategy with two other genetic control strategies that have been developed in *Ae. aegypti*: release of a strain with female-specific lethality, and a strain with both female-specific lethality and an anti-pathogen gene. We find that releasing mosquitoes carrying only an anti-pathogen construct can substantially decrease vector competence of a natural population, even at release ratios well below that required for the two currently feasible alternatives that rely on population reduction. Finally, although current genetic control strategies based on population reduction are compromised by immigration of wild-type mosquitoes, releasing mosquitoes carrying only an anti-pathogen gene is considerably more robust to such immigration.

**Conclusions/Significance:**

Contrary to the widely held view that transgenic control programs aimed at population replacement require linking an anti-pathogen gene to selfish genetic elements, we find releasing mosquitoes in numbers much smaller than those considered necessary for transgenic population reduction can result in comparatively rapid and robust population replacement. In light of this non-intuitive result, directing efforts to improve rearing capacity and logistical support for implementing releases, and reducing the fitness costs of existing recombinant technologies, may provide a viable, alternative route to introgressing anti-pathogen transgenes under field conditions.

## Author Summary

Dengue is transmitted by the *Aedes aegypti* mosquito. Releases of genetically sterile males have been shown to reduce wild mosquito numbers. An alternative approach is to release mosquitoes carrying genes blocking dengue transmission. It is often assumed that spreading such genes in mosquito populations requires using selfish genetic elements (SGEs - genes that are inherited at higher rates than other genes in the genome). Absent such techniques, the release numbers required to transform mosquito populations is seen as prohibitive. However, strategies that rely on SGEs or related technologies to spread anti-dengue genes are unlikely to be implemented in the near future as a public health response. Using a biologically detailed model of *Aedes aegypti* populations dynamics and genetics, we assess how many mosquitoes need to be released to spread an anti-pathogen gene in an urban environment without using an SGE. We compare release numbers with two other, currently feasible transgenic strategies: releasing mosquitoes with female-lethal genes, and mosquitoes carrying both female-lethal and anti-pathogen genes. We show that even without using SGEs, releasing mosquitoes in numbers much smaller than those considered necessary for transgenic population reduction can effectively reduce the ability of *Aedes aegypti* to spread dengue.

## Introduction

The mosquito-borne dengue virus, transmitted primarily by the yellow fever mosquito *Aedes aegypti* (Linnaeus) is estimated to cause approximately 390 million infections each year ([1]). No widespread prophylactic treatments are currently available for dengue, and thus vector population control remains the primary public health strategy. The release of genetically modified mosquitoes provides one approach towards vector population control. Releasing mosquitoes that carry transgenes rendering them vector-incompetent could, in principle, facilitate the prevention of epidemics by replacing wild-type populations of *Ae. aegypti* with mosquitoes carrying transgenic anti-pathogen constructs (e.g., [2]). Ultimately, the frequency of such anti-pathogen transgenes must increase to fixation (e.g., [3]), or at least reach and remain at a sufficiently high frequency to lower the number of competent vectors to levels preventing epidemic outbreaks (e.g., [4], [5]).

*Aedes aegypti* populations in cities are potentially quite large, and the large release numbers generally perceived to be necessary for population replacement has inspired the development of several transgenic gene-drive strategies and related approaches. These approaches including linking anti-pathogen constructs to MEDEA ([6]) or homing endonuclease genes ([7] and [8]), inducing widespread *Wolbachia* infections (e.g., [9], [10] and [11]), among others (reviewed in [3] and [12]). These gene-drive strategies vary in their stages of development and future potential for application in public health contexts. Only those based on *Wolbachia* symbionts have thus far been demonstrated to be workable in the field for mosquitoes ([13]). Even for *Wolbachia*-based interventions, the epidemiological impact of this approach under realistic conditions remains unknown. For instance, the epidemiological performance of *Wolbachia*-induced refractoriness, particularly in regions where multiple serotypes potentially circulate (e.g., [14] and [15]) remains to be tested.

Introducing anti-pathogen genes into wild populations therefore remains a major challenge. Moreover, because any specific anti-pathogen gene for reducing disease transmission in the field could fail due to pathogen resistance evolution, a system that enables effective replacement of one anti-pathogen transgene with another is critical to the long-term success of population replacement programs. As none of the proposed transgenic anti-pathogen gene-drive mechanisms are likely to be implemented as public health measures in the near future ([16], [12] and [17]), transgenic strategies aimed at suppressing mosquito populations have received considerable attention ([18], [19], [20], and [21]). These approaches are inspired by the sterile-insect technique (SIT), whereby a large number of sterile males are reared, irradiated and released, subsequently mating with wild females and thereby reducing the fecundity of wild female mosquitoes ([22], [20]). Although traditional SIT programs for *Ae. aegypti* have never been implemented over large geographic regions or for extended periods of time ([23] and [24]), transgenic population reduction strategies in *Ae. aegypti* have advanced to field trials ([25] and [26]). Our examination of the literature indicates that there is a prevailing view that population suppression by repeated release of transgenic, sterile mosquitoes may be practical ([18], [24]). By contrast, aiming at population replacement by repeated release of mosquitoes carrying a single anti-pathogen gene without a gene-drive mechanism is generally not seen as practical (e.g. [3], [27] and [28]), unless the transgenic construct can provide a net fitness benefit to refractory mosquitoes (e.g., when the virulence of the pathogen to mosquitoes is high - [29] and [30]).

Yet despite this prevailing view, there are surprisingly few quantitative assessments of how releasing anti-pathogen genes into a mosquito population without an accompanying gene-drive mechanism could alter the genetic composition of natural *Ae. aegypti* populations. Quantifying the effectiveness of release strategies aimed at population replacement that do not rely on gene-drive mechanisms under realistic conditions is key to establishing a baseline against which the efficacies of more elaborate drive mechanisms can be compared ([29]). Such an assessment can potentially help evaluate the performance of existing anti-pathogen genes under field conditions (e.g., [27]), as well as identify key facets of mosquito biology that might promote or hinder the spread of anti-pathogen constructs in the absence of gene-drive (e.g., mating preferences among transgenic and wild-type mosquitoes - [30]).

Here we use Skeeter Buster ([31]), a biologically detailed model of *Ae. aegypti* population dynamics parameterized for a tropical city, to quantitatively assess whether a transgenic control program based on rearing and releasing transgenic *Ae. aegypti* carrying a single anti-dengue gene (e.g., [2]) could provide a feasible approach to population replacement in the absence of gene-drive mechanisms. We compare our results to release sizes necessary for suppressing vectoring mosquito numbers with a transgenic strain carrying 1) a single anti-pathogen gene, 2) a single female-killing gene, and 3) both an anti-pathogen gene and a female-killing gene (e.g., [32]).

## Materials and Methods

### Model description

Skeeter Buster models the population genetics and dynamics of *Ae. aegypti* in an urban setting, incorporating key processes in the life cycle, including temperature-dependent survival rates, container-level development and nutrient dynamics, oviposition, and dispersal ([31]). Four life stages are explicitly modeled: eggs, larvae, pupae, and adults. Development, reproduction and mortality are stochastic, but these processes are also driven by temperature- and, for development, resource-dependent rates. Skeeter Buster models individual water-holding containers located in specific houses (“sites”) laid out on a rectangular grid. Resource dynamics within containers and the feedback between larval biomass and resource availability (i.e., density dependence) in containers with mosquitoes in them are based on the equations used in [33]. Mosquitoes emerge as adults and occupy sites where the containers in which they developed are located. Females select mates among males in the same site, mate only once during their lives, and oviposit in containers at the site they occupy on a given day during each gonotrophic cycle. Adults can potentially migrate each day to a randomly selected adjacent site (fixed daily probability of migration  = 0.3 based on the mark-release recapture studies of [34] and parameterized according to [31], [35]). In Skeeter Buster, females may also undergo occasional long range dispersal events (for instance, via inadvertent translocation in vehicles). Such long distance dispersal is modeled by allowing a small proportion (2%) of individuals to disperse to a site randomly selected within a Manhattan distance of twenty sites. The resulting dispersal pattern has been shown to be consistent with field studies (e.g., [36] and [31]). For a more thorough description and justification of the features and components of the model, see [31], [35], and [37].

Because Skeeter Buster aims to model the biology of *Ae. aegypti* in a realistic environment, it must be parameterized in a location-specific manner with local meteorological data and a description of the distribution of containers. Data from the equatorial Amazonian city of Iquitos, Peru (73.2W, 3.7S), which has been subject to long-term, detailed larval habitat surveys (e.g., [38]), has proven particularly well-suited for use with Skeeter Buster (e.g., [31], [35] and [37]). Although there is currently no planned release of transgenic mosquitoes in Peru (A. Morrison, pers. comm.), using Iquitos as a case-study allows us to build on this earlier work and apply Skeeter Buster to model the population dynamics of *Ae. aegypti* at this location (e.g., [37]). As in [39], a 2448 house subsection (68 houses ×36 houses) of the city is simulated with periodic boundary conditions, following the approach described in [37]. In Iquitos, the average distance between sites is on the order of 5-10 meters ([37]), and the simulated region corresponds to an area of approximately one square kilometer of a densely populated neighborhood in Iquitos. These conditions result in an equilibrium population size of approximately 14000 total female adult mosquitoes (or approximately 6 female adult mosquitoes per site) prior to releases of transgenic mosquitoes.

Skeeter Buster follows the genotypes of mosquitoes, allowing evaluation of changes in the genetic composition of the population that result from releasing transgenic mosquitoes. This feature permits quantitative comparisons between our results and those obtained for alternative genetic control strategies. The anti-pathogen and female-specific lethal genes are both modeled as diallelic loci. Each locus is characterized by the presence or absence of the transgenic construct. For brevity, we refer to individuals that lack both constructs as “wild-type mosquitoes”. We simulate introgression by the anti-pathogen transgene when mosquitoes carrying only the anti-pathogen gene are released (for brevity, we denote this as the “AP” strategy). We compare the resulting reduction in competent vectors to reductions caused by a transgenic control program based on the female-specific lethal gene alone (an “FK” strategy aiming exclusively at population reduction), as well as to a “reduce and replace” (“RR”) strategy based on releasing mosquitoes carrying both the anti-pathogen and female-specific lethal transgenes. As an FK strategy permits the introduced female-specific lethal transgene to remain in the population past the first generation, it can potentially accelerate population reduction relative to non-sex specific lethal constructs (e.g., [40]). To highlight the contrast between transgenic strategies based on population reduction and approaches aiming at population replacement, we do not analyze population reduction strategies that cause both male and female offspring mortality (for instance, bisex RIDL and conventional SIT strategies - e.g., [20] and [25]). The female-specific lethal gene is assumed to be expressed only when eggs are reared in the absence of tetracycline ([19]).

Inserting transgenes into the *Ae. aegypti* genome may potentially disrupt the normal functioning and regulation of the inserted site. We assume such fitness costs may reduce the expected survivorship of transgenic individuals relative to wild-type individuals by 5-10% per copy of the anti-pathogen transgene carried (the midpoint and upper limit of the range used in, e.g., [27] and [41]). For simplicity, and to facilitate our comparison of the different strategies, we do not model any fitness costs associated with the female-specific lethal gene aside from female-specific conditional mortality. We also assume that when present, costs associated with the anti-pathogen gene are expressed at the egg stage for both male and female mosquitoes, and hence potentially result in a reduction in larval production when either parent carries a transgene copy ([42]). We compare how the presence and absence of such fitness costs associated with the transgene insertion affect prospects for population replacement. While the dengue virus may confer a fitness cost on infected mosquitoes (e.g., [43]), how much evading infection facilitates the transgene's spread depends, in part, on dengue prevalence among vectors ([29]). This prevalence, in turn, can be highly location- and time-specific, and governed by several factors including local epidemiological dynamics, the susceptibility of mosquitoes to vertical transmission of dengue, as well as potentially seasonal effects (e.g., [44], [45] and [46]). Incorporating all these processes into our model is beyond the scope of our study. Thus, for simplicity and to stringently test the effectiveness of population replacement strategies absent gene-drive, we assume throughout that mosquitoes carrying the anti-pathogen gene enjoy no fitness benefit relative to wild-type mosquitoes.

Finally, genetic control programs relying on population reduction alone are vulnerable to reinvasion if releases cease. Wild *Ae. aegypti* individuals from populations that have not been subject to control efforts can immigrate into the population, undermining sustainable population reduction in the absence of ongoing releases ([47] and [48]). By contrast, the effects of immigration on the efficacy of population replacement strategies are relatively understudied. [39] shows how an RR strategy can also be subject to such adverse effects of immigration. Comparing the robustness of the different strategies in the face of immigration by wild-type mosquitoes can therefore be critical in evaluating their potential under biologically relevant conditions. We thus assess the impact of gravid wild-type immigrant females on the efficacy of the population replacement programs we consider.

### Modeling the release regimes

All released transgenic individuals are modeled as homozygotes carrying two copies of the anti-pathogen transgene. The anti-pathogen gene is considered to have dominant phenotypic expression. Because transgenic female mosquitoes carrying an anti-pathogen gene do not transmit dengue, we compare male-only adult releases (which may be more readily amenable to regulatory and community approval e.g., [49] and [50]) to bi-sex adult releases that would increase nuisance biting to some extent. Releases of genetically-modified mosquitoes are modeled by dynamically adding cohorts of mosquitoes homozygous for the anti-pathogen gene at specified dates to specific sites following a single burn-in year permitting the population to attain demographic equilibrium (e.g., [51] and [39]).

Distributing and releasing transgenic adults can be logistically challenging. For instance, the adult-stage of *Ae. aegypti* is comparatively short-lived in the field, and thus releasing adult mosquitoes may require planning releases to coincide with the emergence of individuals as adults. An alternative strategy is to release transgenic mosquito eggs. *Ae. aegypti* eggs are desiccation-resistant and can survive for extended periods of time (e.g., [52], [53]), permitting long-term storage and subsequent distribution. Using viable eggs has therefore been suggested as an alternative to releasing adults ([54], [24] and [51]). Eggs could also potentially be easier to handle, reducing the costs associated with transporting transgenic strains. We therefore evaluate the effectiveness of a release program involving the distribution of transgenic eggs instead of adults. Following [19], [51] and [39], the release of eggs is modeled as weekly additions into specified sites of nutrient-filled containers shielded from ovipositing females. We model 100 to 1600 eggs placed in covered, nutrient-filled containers. Although sufficient resources are provided in the containers, only approximately 40% of the eggs ultimately develop to adulthood due to natural mortality. These added containers are removed from the simulation upon the emergence as adults or death of all mosquitoes they contain. Because male and female eggs cannot currently be separated, an equal number of eggs of both sexes are assumed to be placed in each of the containers.

We base the release ratios for mosquitoes on those used in an earlier study ([51]) employing Skeeter Buster to evaluate how releasing transgenic mosquitoes carrying a single conditionally-lethal construct could facilitate population reduction. The largest release numbers for which we present results (totaling approximately 120,000 adult males per week) corresponds to a release ratio of approximately 17.5∶1 released males to wild-type males present when releases begin. These ratios are within the range used in control programs for mosquitoes based on the sterile insect technique (e.g., [55]).

[51] found that the weekly release numbers necessary to cause local population extinction depended on the geographic penetrance of the release regime. Furthermore, the ability to implement the transgenic control programs characterized above could also depend on the accessibility of different sites in the urban arena. Release sites may be clumped in space in some instances, whereas in other cases it may be feasible to release individuals at regularly-spaced points across the grid, or, through aerial releases of adults, potentially into a very high proportion of sites. To approximate the potential effect of different spatial implementations, we model three idealized spatial configurations: homogenous releases everywhere, regular point-source releases at every 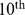 site at regularly-spaced points in the grid, and random point-source releases at 10% of the sites randomly chosen. None of these regimes can be expected to be achieved exactly under field conditions, but comparing the distinct spatial patterns can help inform the spatial configuration of releases that actual transgenic control programs should strive to reproduce. For the operational reasons explained in [51], we do not examine the distribution of eggs into every site. In the cases where releases only occur in a subset of sites, we fix the sites at which releases take place for the duration of each simulation run. If the composition of release sites changes frequently as the releases are carried out, then the release regime can be expected to begin to approximate the homogenous configuration over time. Such homogenization can make it difficult to contrast the effects of releasing transgenic mosquitoes at a limited number of sites to the case where releases occur at all sites.

Following [51] and [39], we assume indefinite releases cannot be sustained. Thus, we compare a relatively short (one year) release period to a longer (three year) release period. To assess the fate of the transgene after releases end, we run the model for an additional two years. Finally, because Skeeter Buster is a stochastic model, we run 30 simulations of each release regime varying the spatial arrangement of sites (and hence containers) in the grid for each simulation. For the random point-source releases, we also randomize the release sites at the beginning of each simulation.

## Results

**Figure 1:**
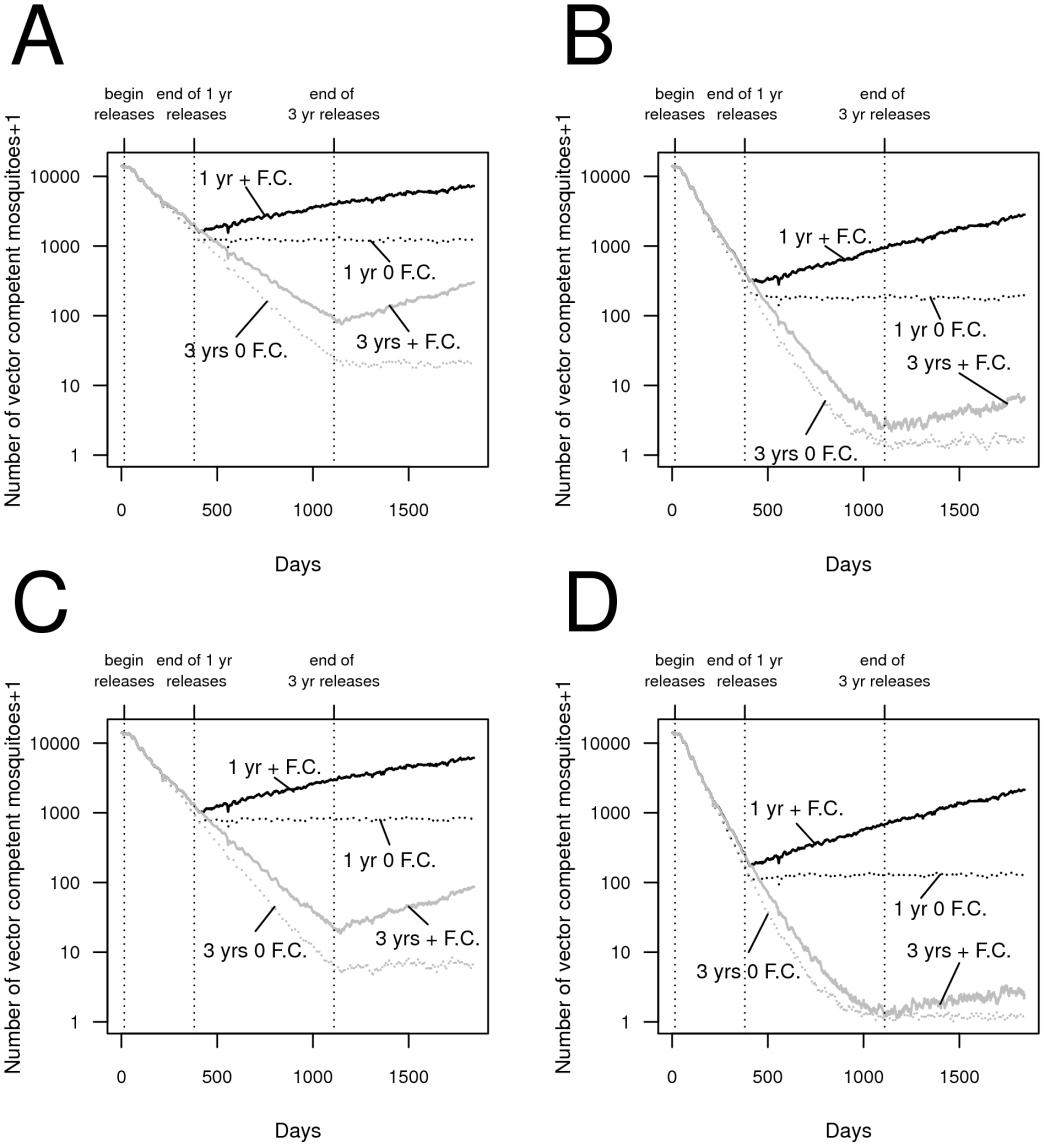
The effect of releasing transgenic mosquitoes carrying a single anti-pathogen gene on the reduction in vector-competent (i.e., wild-type adult female) mosquitoes in the population. (A) Unisex releases of 20 adult males per site per week at 10% of the sites regularly spaced apart, when releases occur over a single year (black lines) and three year (grey lines) period, respectively, in the presence (solid lines) and absence (dashed lines) of a fitness cost, (B) bisex releases (with a 1∶1 sex-ratio) of 10 male and 10 female adult mosquitoes per site per week, when releases occur at 10% of the sites regularly spaced apart over a single year (black lines) and three year (grey lines) period, in the presence (solid lines) and absence (dashed lines) of a fitness cost, (C) unisex releases of 2 adult males per site per week, when releases occur at all sites over a single year (black lines) and three year (grey lines) period, respectively, in the presence (solid lines) and absence (dashed lines) of a fitness cost, (D) bisex releases (with a 1∶1 sex-ratio) of 1 male and 1 female adult mosquito per site per week, when releases occur all sites over a single year (black lines) and three year (grey lines) period, in the presence (solid lines) and absence (dashed lines) of a fitness cost. Increasing the release duration reduces the vector competent population substantially. The time series represent the averages over 30 runs; in this, and in subsequent figures, “+F.C.” refers to an anti-pathogen construct bearing a fitness cost of 5% per copy unless stated otherwise, and “0 F.C.” refers to releases using constructs that do not carry a fitness cost. Here, and in subsequent figures, as the vertical axes are plotted on a log scale adding 1 to the average number of competent vectors allows depicting the elimination of competent vectors on this scale. Dynamics from the burn-in periods are not shown.

We find that even relatively modest release numbers of transgenic mosquitoes carrying a single anti-pathogen construct can reduce the abundance of vector-competent mosquitoes within the first year of releases, in some cases to very low levels (Fig. 1). Continued releases occurring over a longer duration lower competent vector numbers much further. If the anti-pathogen gene is not fixed, a fitness cost associated with the anti-pathogen gene can slow the rate of population replacement as releases occur (e.g., Fig. 1A) and allows the reemergence of competent vectors. When releases can drive the number of vector competent mosquitoes to very low levels, the rate of recovery of the vector-competent population after releases end is modest with more than 90% of the vector population still incompetent two years after releases end (e.g., Figs. 1B, D). Bi-sex releases of adults can cause greater reductions in vector competence compared to similarly-sized male-only adult releases (Figs. 1A and 1C versus Figs. 1B and 1D). Finally, we find the vector competent mosquito population declines further when adults are released at all sites compared to when adults are released in every 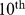 site (e.g., Fig. 1A versus Fig. 1C).

**Figure 2:**
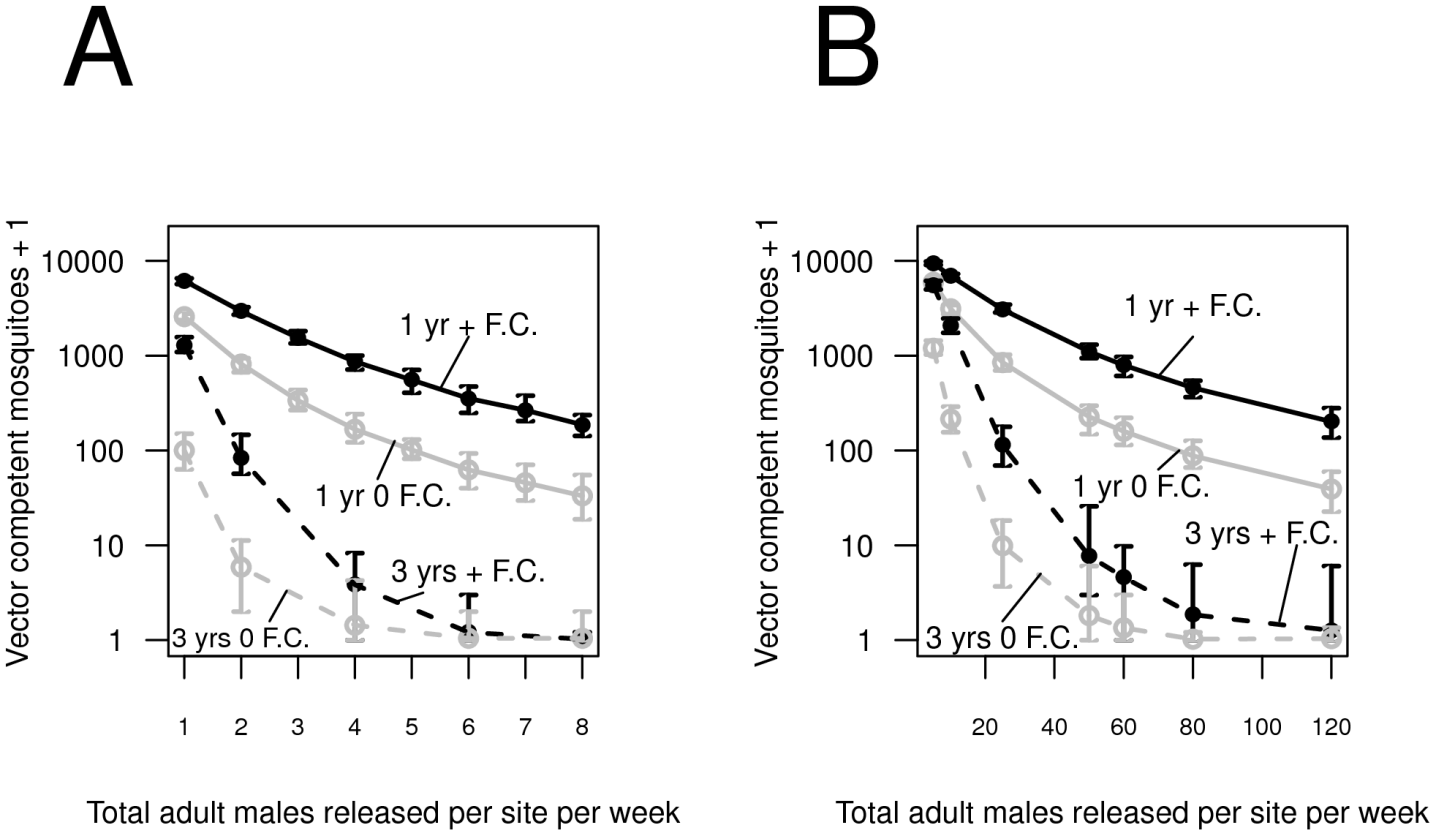
The average reduction in vector-competent females from 30 simulation runs two years after releases end for male-only releases (A) at all sites and (B) at 10% of the sites laid-out across a regular grid with (black lines) and without (grey lines) a 5% fitness cost associated with the anti-pathogen transgene. Solid lines represent releases for a single year, and dashed lines represent releases for three years. For transgenic control programs lasting a single year, between 130,000 and 3 million total mosquitoes are released, while for programs lasting for three years, between 380,000 and 9 million total mosquitoes are released. The end points on the error bars represent the 2.5th and 97.5th percentile abundances across simulation runs.

**Figure 3:**
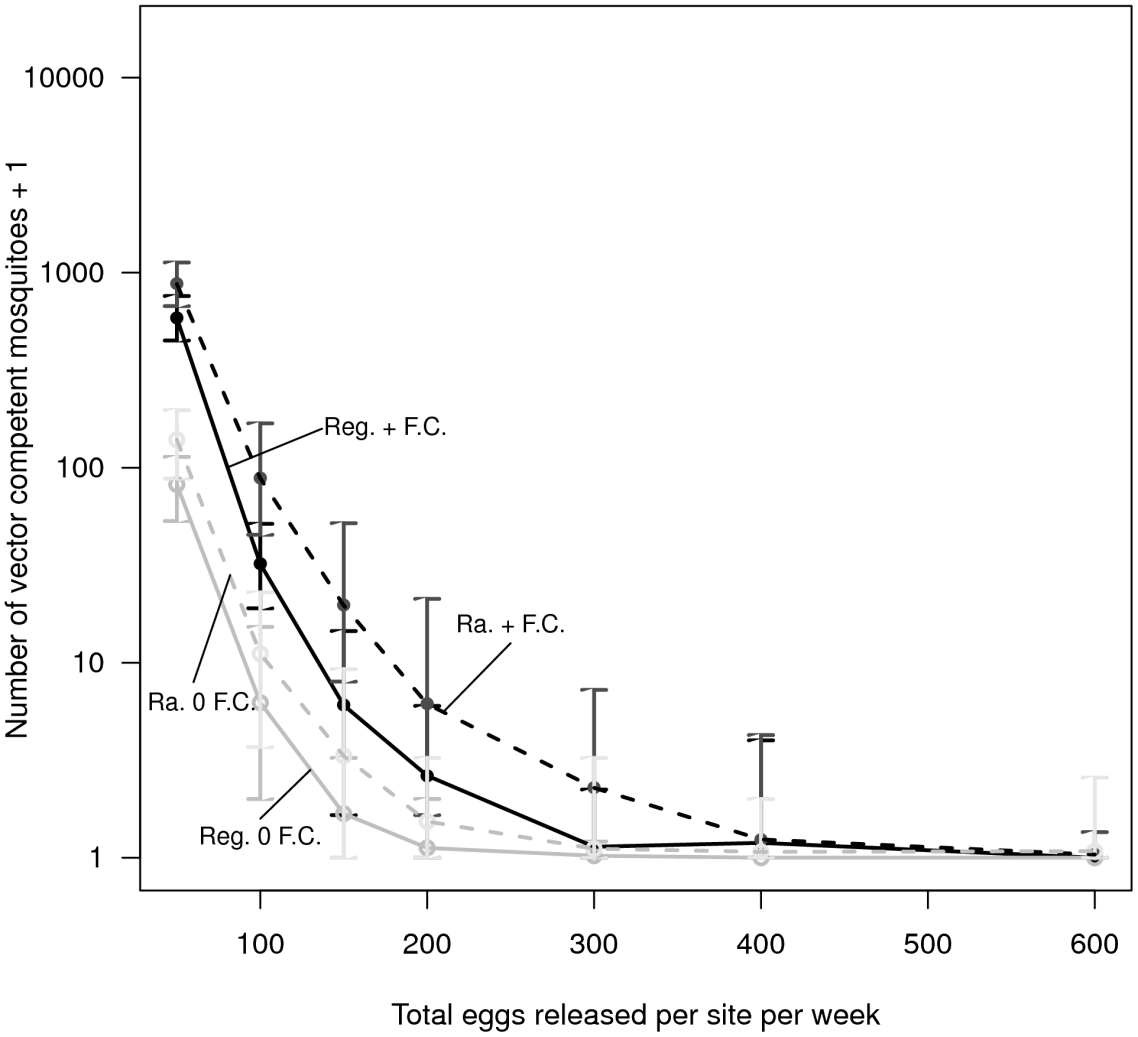
The average reduction in vector-competent females from 30 simulation runs two years after releases end across release numbers when different numbers of transgenic mosquito eggs (of both sexes) are released at 10% of the sites along a regular grid (Reg.) for a single year (solid lines), and at 10% of the sites selected at random (Ra.) for a single year (dashed lines). Black lines represent results in the presence of a fitness cost associated with the anti-pathogen transgene, and grey lines represent the results assuming no fitness cost. For transgenic control programs lasting a single year, approximately 650,000 to 7.7 million total eggs are released, while for programs lasting for three years, approximately 190,000 to 23 million total mosquito eggs are released. Although sufficient resources are provided in the containers, only approximately 40% of the eggs ultimately develop to adulthood due to natural mortality. As in Fig. 2, the end points of the error bars represent the 2.5th and 97.5th percentile abundances across simulation runs. Solid error bars correspond to releases along a regular grid, and translucent error bars correspond to releases at a random subset of sites.

Although the release regimes we analyzed can cause substantial reductions in the population of competent vectors, increasing the number of mosquitoes released is key to driving the number of vector-competent female mosquitoes to very low numbers two years after releases end (Figs. 2–3). Increasing this release number can compensate for the detrimental effect of the fitness cost associated with the anti-pathogen gene. For the same number of total mosquitoes released throughout the release program, multi-year releases can be more effective than single year releases (Fig. 2). For instance, releasing two males each week at all sites for three years causes a greater reduction in the abundance of vector competent mosquitoes compared to releasing six males at all sites for a single year. Furthermore, if releases are spatially homogenous, fewer total males need to be released compared to male releases at every 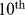 site, but both spatial release strategies permit substantial population replacement when the total number of released males is approximately 20000 males per week across the entire neighborhood (Fig. 2A v. Fig. 2B).

We also note that, when both male and female mosquitoes can be released, many fewer total mosquitoes need to be released every week compared to unisex releases to facilitate replacement (Figure S1).

Because the containers with transgenic eggs permit the emergence of transgenic mosquitoes of both sexes, releasing as few as 100 eggs per week per house in 10% of houses can cause substantial population replacement (Fig. 3), reducing the population of vector competent females to below 10% of pre-release levels in a single year. Increasing the spatial regularity of releases facilitates long-term replacement (Fig. 3). For the same number of eggs distributed, reducing the fitness cost lowers the number of vector competent mosquitoes two years after releases end by almost an order of magnitude (Fig. 3).

### Comparison to transgenic control strategies involving reduction

**Figure 4:**
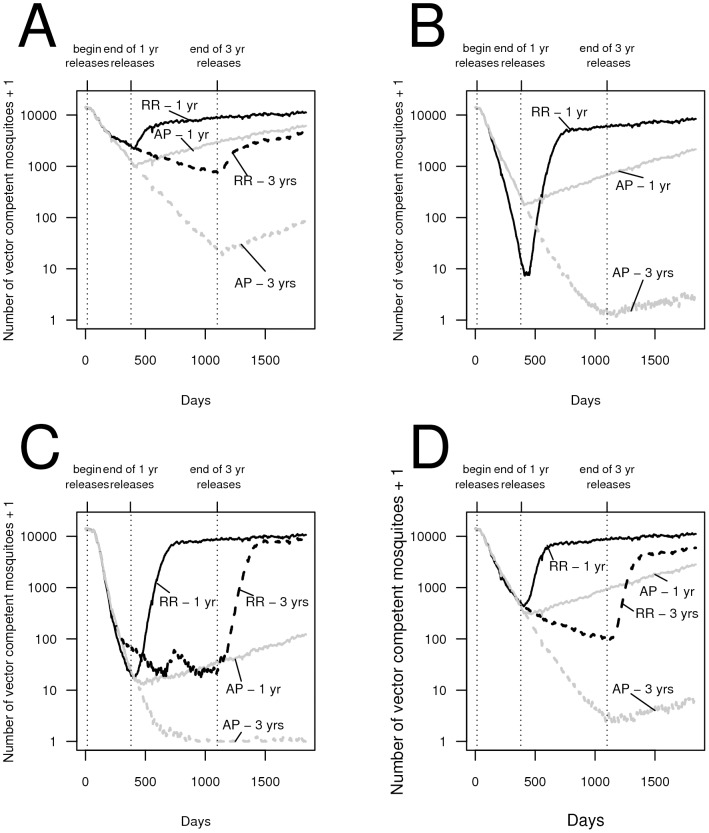
The average number of vector competent mosquitoes for each day for up to 30 simulation runs modeling “reduce and replace” (RR) transgenic control strategies and a strategy releasing mosquitoes carrying the anti-pathogen construct alone (an AP strategy). (A) adult males are released at all sites, (B) adult male and adult females are released at all sites, (C) eggs are released in 10% of the sites along a regular grid, and (D) adult males and females are released in 10% of the sites along a regular grid. The numbers of mosquitoes released per site for the illustrated time series are: (A) 2 adult males, (B) 1 adult male and 1 adult female, (C) 100 eggs, (D) 10 adult males and 10 adult females. In this, and in subsequent figures, we assume that the conditionally-lethal construct carries no additional fitness costs beyond dominant female adult lethality. In all panels, solid lines represent releases for a single year and dashed lines represent 3 year releases. RR releases are illustrated in black and AP only releases are in grey. Lines from release scenarios where all vector competent mosquitoes were eventually eliminated (either through population extinction or complete replacement) in all runs are omitted, and thus not all 30 simulation runs are included in calculating the average numbers for the RR strategy.

Across all release scenarios, releases of transgenic mosquitoes under a “replacement-alone” (AP) strategy (mosquitoes carrying an anti-pathogen gene without a conditionally-lethal construct) lowered the long-term population of vector-competent mosquitoes more successfully than the “reduce and replace” (RR - mosquitoes carrying both the anti-pathogen and female-lethal construct) strategy (Fig. 4). In comparison to the RR releases, even in the presence of a fitness cost, releases of mosquitoes under the AP strategy proved capable of maintaining the vector-competent population at low levels. We note that these simulations assume the female-specific lethal construct provides no additional fitness cost (e.g., male carriers of the lethal gene are unaffected). When the female-killing transgene is likely to carry to a fitness cost, the reduction in competent vectors for the RR (and FK) strategy may be considerably less than what we show here (e.g., [51]).

The contrast between the two transgenic control strategies can be particularly pronounced when releases last for three years and the anti-pathogen gene carries a fitness cost. For an RR strategy, the detrimental effects of a fitness cost can become more apparent especially during population recovery as the entire mosquito population grows rapidly from low numbers. This results in the competent vector population size quickly recovering towards pre-release levels. By contrast, even in the presence of a fitness cost, releases of mosquitoes carrying only the anti-pathogen gene result in low numbers of vector-competent mosquitoes for extended periods of time following the end of releases, and the vector competent population recovers much more slowly. We also find transgenic control strategies based on population reduction alone (the FK strategy) fail to lower the long-term vector competent population sizes below the levels obtained using the RR and AP strategies, even when the anti-pathogen construct carried a fitness cost (Figure S2).

**Figure 5:**
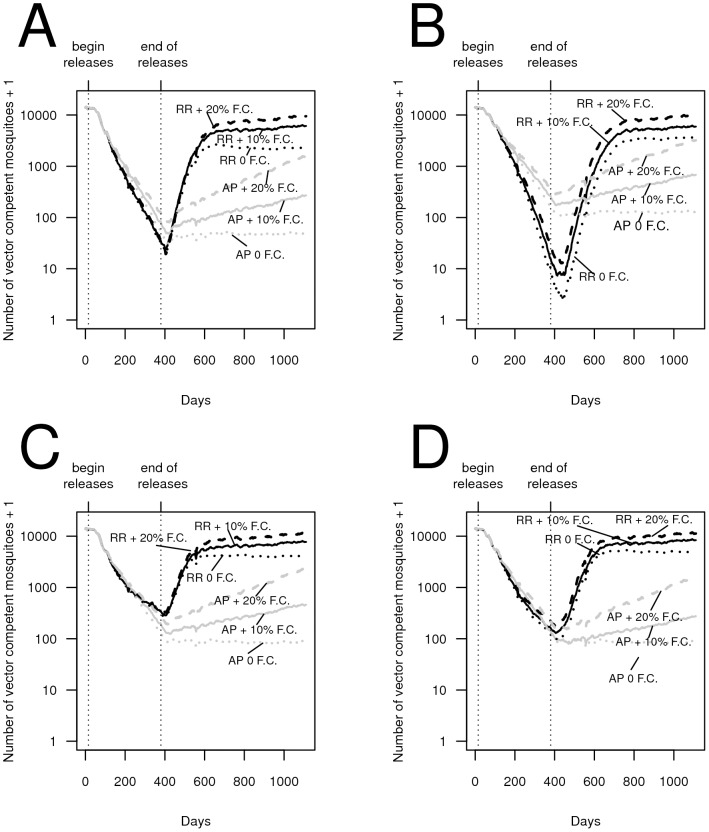
The average number of vector competent mosquitoes for each day for up to 30 simulation runs modeling “reduce and replace” (RR) transgenic control strategies and a strategy releasing mosquitoes carrying the anti-pathogen construct alone (an AP strategy) under differing fitness costs associated with the anti-pathogen gene. In all panels, dotted lines represent the absence of a fitness cost associated with the anti-pathogen gene, solid lines represent a fitness cost of 5% per copy of the anti-pathogen gene, and dashed lines represent a fitness cost twice the value used in previous figures (10% fitness cost per copy). RR releases are illustrated in black and AP-only releases are in grey. The results are for (A) adult male releases at all sites, (B) adult male and adult female releases at all sites, (C) adult male releases at 10% of the sites along a regular grid, and (D) adult male and adult female releases at 10% of the sites along a regular grid. We illustrate the dynamics from a single year of releases with per-site release numbers of (A) 7 adult males, (B) 1 adult male and 1 adult female, and (C) 80 adult males, and (D) 15 adult males and 15 adult females. Lines from release scenarios where all vector competent mosquitoes were eventually eliminated (either through population extinction or complete replacement) in all runs are omitted.

The AP strategy consistently lowered the long-term number of vector-competent mosquitoes more than the RR strategy. Under an RR strategy, population reduction can release the surviving mosquitoes from density-dependent constraints, allowing surviving wild-type mosquitoes to have high per-capita growth rates. By contrast, the AP strategy does not lower population density, and hence density dependence can remain strong even as releases are ongoing. Under some conditions (e.g., when only a small number of adult males are released Fig. 4A), this difference between the two strategies prevents the RR strategy from being able to lower the number of vector competent mosquitoes as effectively as the AP strategy once the vector competent population has been reduced, even when releases are ongoing (e.g., Figs. 4A, 4C, and 4D). Thus, for some release scenarios (e.g., releases of eggs), an AP strategy can have a larger transient effect on vector-competent population reduction than an RR strategy (e.g., Fig. 4C and Fig. 4D). By contrast, when the RR strategy is comparatively effective at reducing mosquito abundances (e.g., when sufficiently large numbers of females are also released - e.g., Fig. 4B), an RR strategy can cause greater reductions in the vector-competent mosquito population during the transient stages in some of the release scenarios (Figs. 4 and 5). We find that, when present, these differences are most pronounced after there has been an approximately three orders of magnitude decline in the vector-competent population caused by an RR strategy, although the difference can be apparent by 200 days into releases (e.g., Fig 5A). When the fitness cost is very high, the difference between an RR strategy and an AP strategy during the transient stages has the potential to be modestly larger than when there is weaker or no fitness cost, because the higher fitness cost renders an anti-pathogen construct less capable of spreading through an AP strategy. An FK strategy based on population reduction alone proves unable to lower the number of vector competent mosquitoes further than the RR strategy, even during the transient stages (Figure S2). These results are robust to whether simulation runs resulting in population extinction are included in the analysis (Figures S3-S4).

### Immigration of wild-type females

**Figure 6:**
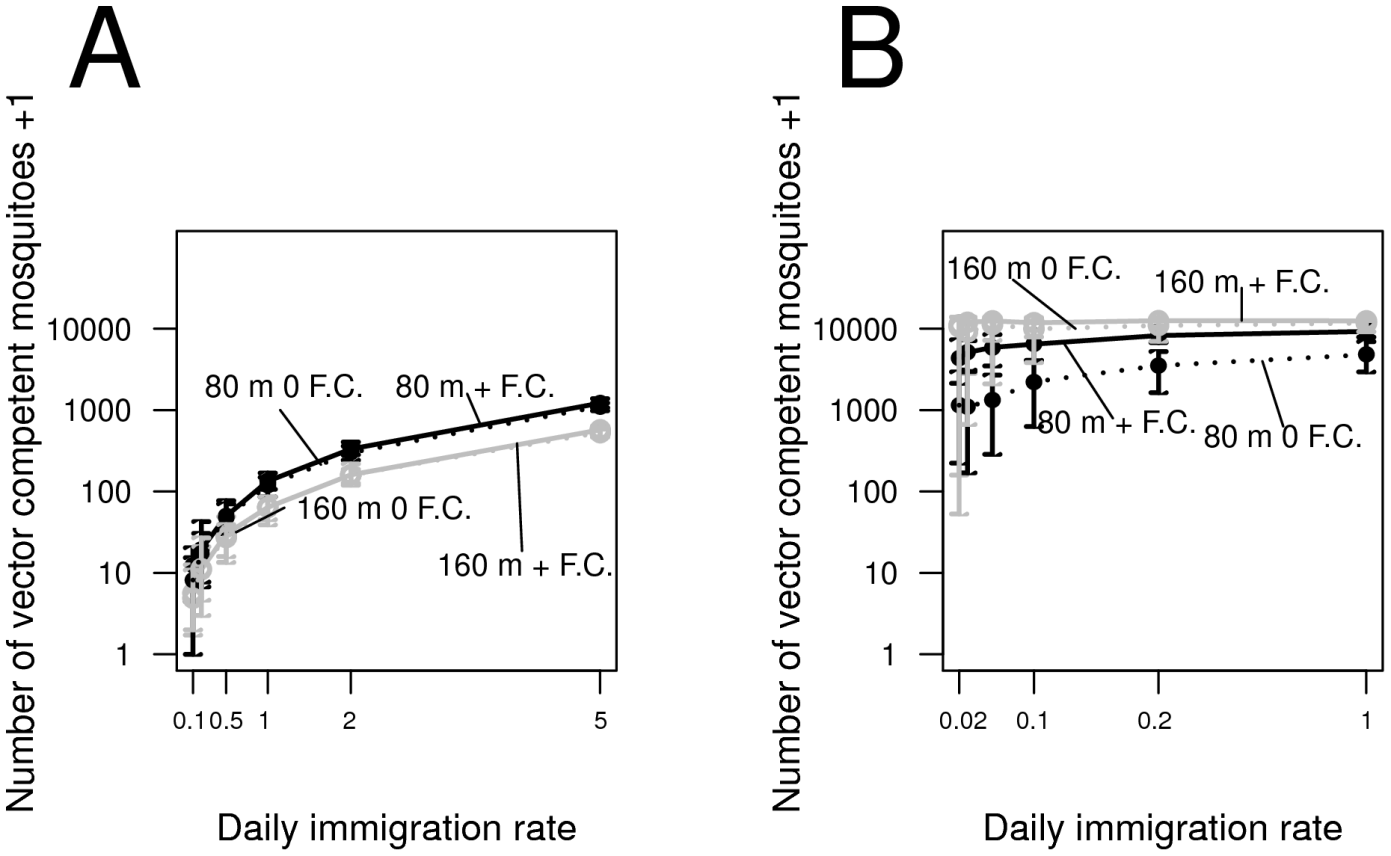
The effect of the daily immigration rate on the number of vector competent mosquitoes two years after releases end under a three year release of adult male transgenic mosquitoes at 10% of the sites along a regular grid when (A) mosquitoes carrying just the anti-pathogen construct are released (an AP strategy) and (B) mosquitoes carrying both an anti-pathogen and female-lethal transgene are released (an RR strategy) with (solid lines) and without (dashed lines) a fitness cost with 80 (black lines) and 160 (grey lines) males released per site. The end points of the error bars represent the 2.5th and 97.5th percentile abundances across simulations. Only gravid, wild-type females carrying neither the anti-pathogen nor the conditionally-lethal gene are assumed to migrate into the urban arena. Such females are assumed to immigrate into any site in the simulated region at random. Under the RR strategy, when the immigration rate is low the combined effects of genetic drift at small population sizes and the spatially heterogeneous nature of the population recovery after releases end amplify the variability across runs in the number of competent vectors (e.g., [39]). Each model run represents a different, randomized spatial configuration of sites into which migrants arrive.

We find that immigration by gravid, mature wild-type females has an appreciable effect on the long-term numbers of vector competent females under an AP transgenic control strategy. As immigration from wild-type populations increases (e.g., at least 5 immigrants per day across the simulated region), the frequency of wild-type mosquitoes can also increase several fold (Fig. 6A). Increasing the number of AP-only mosquitoes released has only a small effect on these results, as does eliminating the fitness cost associated with the anti-pathogen transgene. As the immigration rate increases, the effect of increasing release numbers on the number of vector competent mosquitoes becomes more apparent (black versus grey lines, Fig. 6A).

Although immigration can potentially increase the number of competent vectors, its impact on transgenic control strategies other than AP can be more pronounced. For instance, under an RR strategy, even comparatively rare immigration events severely undermine population replacement efforts (Fig 6B; see also [39]). Moreover, when the transgenic control strategy involves population reduction, increasing the numbers released can actually accentuate the effect of wild-type immigration events (Fig 6B; see also [39]).

## Discussion

Because the genetic engineering of anti-pathogen constructs for *Ae. aegypti* is somewhat further developed than for gene-drive mechanisms (e.g., [27], [12] and [17]), it is critical to assess the prospects of alternative approaches to spreading currently proposed anti-pathogen constructs under field conditions. Such an assessment could provide a baseline against which the field efficacies of gene-drive mechanisms and related approaches could be compared (e.g., [29]). As genetic control methods based either in whole or in part on population reduction may require considerable resources to sustain, strategies aimed at population replacement provide an alternative approach to achieving long-term reductions in vector competence. Based on simulations with a stochastic, spatial model of a natural population of *Ae. aegypti*, we find that releasing mosquitoes carrying only a single anti-pathogen construct at ratios well below those considered necessary for transgenic technologies based on population reduction can facilitate robust reductions in vector-competence in a reasonable time frame, in some cases reducing the average number of competent vectors to between 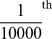 to 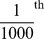 of pre-control levels (e.g., Figs. 1 and 2). These reductions compare favorably to reductions in vector capacity considered necessary to achieve public health goals. For instance, reducing vector capacity (as measured via house indices) using source removal, space spraying and legal and educational interventions to between 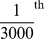 to 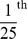 of pre-control levels in Cuba and Singapore facilitated dengue control in both countries in the 1980s ([56]). However, we caution that the ultimate epidemiological benefits of any transgenic control program depends on the effectiveness of the anti-pathogen transgene under field conditions.

When transgenic females are unable to carry dengue, we also find that releasing females in addition to males greatly reduces the number of mosquitoes necessary to reduce vector competence (Figure 1, Figure 3 and Figure S1). These results appear robust across a range of release regimes. In particular, we also show that releasing even very few eggs per house (especially in comparison to the number of eggs that may need to be distributed to cause population elimination - e.g., [51]) to be quite effective. A transgenic control program based on distributing eggs will not require timing release events to coincide with adult emergence events, and may be logistically easier to implement or prove more cost effective. Nevertheless, improving the geographic uniformity of releases for both adult and egg releases facilitates introgression, particularly when transgenic mosquitoes bear fitness costs. Finally, our comparison to other genetic control strategies shows that an AP strategy is considerably more robust to immigration than the RR strategy. Under the RR strategy, increasing release numbers results in a trade-off between population replacement and vulnerability to immigration; by contrast, an AP strategy implies no such trade-off, and the effects of wild-type immigration can be reduced by releasing more mosquitoes carrying only an anti-pathogen construct.

A frequently cited limitation to successfully introgressing an anti-pathogen gene (e.g., [2]) without an accompanying gene-drive mechanism is that such an approach may require prohibitively large release numbers (e.g., [57], [3], [27], [58], [12]). However, based on our results modeling the *Ae. aegypti* population in a neighborhood of approximately 2500 houses in Iquitos, Peru, total weekly release numbers of less than 25000 adult male and female mosquitoes into the expected population of approximately 21000 adults suffice to severely reduce the number of vector-competent females two years after releases end. The total release numbers we simulate are comparable to the number of mosquitoes used to establish *Wolbachia* in the trial studies in Yorkey's Knob and Gordonvale, Australia, communities of approximately 615 and 670 houses, respectively ([13]). There, between 10,000–22,000 *Wolbachia*-infected mosquitoes were released weekly into a seasonal mosquito population ([13]) (or roughly 50–110 mosquitoes per hectare per week, assuming a combined release area of approximately 200 hectares across the municipalities – Text S1). In seasonal environments, such as the communities where the *Wolbachia*-infected mosquitoes were released, releasing mosquitoes as the population is increasing from a seasonal minimum could improve the efficacy of transgenic control strategies using *Wolbachia* ([59]). Yet even for a relatively stable, non-seasonal mosquito population (as in Iquitos), our results suggest that the release densities required for a successful AP strategy may be quite modest. Assuming an average household size of 5.8 people in Iquitos (e.g., [60]) and a land area of approximately 78,400 hectares for the entire city ([61]), our simulated region represents an area of Iquitos containing roughly 4% of the human population. A crude extrapolation of our results suggests that approximately 650,000 mosquitoes would need to be released weekly (for a total of approximately 34 million individuals released per year) throughout an entire city the size of Iquitos to render a substantial fraction of *Ae. aegypti* vector-incompetent at the end of the release duration. This translates into a release density of 8 to 9 released mosquitoes per hectare, per week. By comparison, a transgenic SIT-based release program that reduced the population size of *Ae. aegypti* at a site in the Grand Cayman Islands by about 80% involved approximately 3.3 million engineered males released in a 23-week period ([25]), or roughly 143,000 male mosquitoes per week. This was the equivalent of about 3150 males per hectare, per week, and [25] note that these release rates constitute the minimum necessary to cause population elimination in the absence of immigration. This represents a figure several orders of magnitude larger than the release numbers that we found necessary for introgressing an anti-pathogen gene without an accompanying gene-drive mechanism in our study. Our results therefore suggest that transgenic control strategies based on population replacement could be plausibly implemented to reduce vector capacity even in the absence of mature gene-drive like technologies.

Our comparative modeling approach allows us to highlight why releasing mosquitoes carrying only an anti-pathogen construct (the AP strategy) can lower vector competence more effectively than approaches based on population reduction (the “reduction-only” FK strategy and a “reduce and replace” RR strategy). Skeeter Buster has previously been applied to evaluate transgenic control strategies that rely on population reduction ([51] for an FK strategy, and [39] for an RR strategy). These studies found that some wild-type genes can be expected to persist due to inherent stochasticity in the simulation runs. [39] show that when mosquitoes that do not carry the anti-pathogen gene are able to persist following large population reductions, genetic drift at small population sizes can hinder the effectiveness of the RR strategy. The effects of genetic drift at small population sizes in the RR strategy can be reflected in the genetic composition following population recovery, rendering sustainable reductions in vector competence using an RR strategy very challenging ([39]). By contrast, the AP strategy does not cause population reduction. Continually releasing mosquitoes carrying an AP transgene can therefore monotonically increase the transgene's frequency (e.g., Fig. 1). Furthermore, the AP strategy does not cause population reduction, and thus wild-type mosquitoes remain subject to density-dependent pressures, reducing their ability to contribute heavily to the genetic composition of subsequent generations. These mechanisms allow the AP strategy to perform better than the RR strategy after releases end under a wide array of release scenarios, and improve the robustness of the AP strategy to immigration in comparison to the RR strategy.

However, we note that, at least during the transient stages, the RR strategy can lower vector competence further in some instances compared to the AP strategy. In our simulations, we find such transient effects to be most apparent when the population of vector-competent mosquitoes has already been reduced substantially. Thus, the public health benefits of higher transient reductions could be limited, while the risk can be much more pronounced, particularly considering the long-term failure in the face of immigration of wild-type mosquitoes. Nevertheless, such enhanced (albeit transient) reductions caused by the RR strategy may justify switching between alternative transgenic strains as releases are ongoing. In a subsequent paper, we analyze whether switching between released strains could exploit transient reductions to maximize the potential for long-term reductions in vector competence (Robert et al., in review).

Our work also provides a framework that allows communities and other stakeholders to assess the benefits and costs of implementing different release regimes and genetic control strategies. We anticipate individuals and entities to differ in their willingness to sustain a prolonged genetic control program of *Ae. aegypti* ([62]). Reductions in vector-competence they consider necessary to reduce transmission of *Ae. aegypti*-vectored diseases may depend on, among other factors, receptiveness to genetically-based control strategies (e.g., [23]) or existing public health strategies (e.g., source removal or clinical interventions) that could complement a genetically-based vector control program. Our approach presents a quantitative basis for characterizing both the anticipated reduction in vector competence and the corresponding release numbers that would be required to attain such reductions. Potentially, the costs of implementing a transgenic release program could then be compared to the costs associated with implementing alternative disease management strategies.

Cost-benefit analyses are particularly critical when releases of transgenic females are being considered. The viability of this approach requires carefully weighing real as well as perceived risks in the affected communities. Female releases may be unacceptable unless the anti-pathogen gene renders its carriers completely vector incompetent. Additionally, in communities at risk for other diseases vectored by *Aedes aegypti*, assessing how effectively the transgene protects against other pathogens (e.g., chikungunya - [63]) will be critical. Such risks must be weighed against the cost savings of working with fewer transgenic mosquitoes. Evaluating the marginal effects of producing additional mosquitoes required for male-only releases is key to such an assessment. Our approach allows comparing the number of transgenic mosquitoes required under male-only and bi-sex releases to obtain a given reduction in vector competence. Communities could then weigh the cost of risk mitigation necessary for female releases (e.g., investing in improved anti-pathogen constructs) against the marginal costs of producing and releasing more mosquitoes required for male-only releases.

Our analyses sought to compare the efficacies of distinct transgenic release strategies in a tropical urban environment. However, we calibrated our model for the *Ae. aegypti* population in Iquitos, Peru, and location-specific assessments should precede implementation of a transgenic vector control program in other localities. Such analyses may suggest that some *Ae. aegypti* populations are less amenable to the transgenic strategies we consider. For example, the distribution of breeding containers in other tropical cities may be more spatially heterogeneous than the distribution in Iquitos ([38] and [64]). Under the prevailing pattern of container distribution in Iquitos, there appears to be little spatial clustering in the predicted distribution of female mosquitoes carrying an anti-pathogen transgene (Figure S5). In other localities, if breeding containers are much less evenly distributed than they are in Iquitos, then the spatial distribution of the anti-pathogen construct may differ. Nevertheless, as [51] note, high levels of spatial heterogeneity can also be expected to reduce the efficacy of transgenic control programs based on population reduction. Thus, how increased spatial heterogeneity differentially affects transgenic strategies based on population reduction and strategies based on population replacement may therefore vary by location. Our framework provides a potential approach to compare such effects across different communities.

Our results may also be applicable should it become necessary to eliminate the transgene (see also [27]) or to prevent its spread. Presumably wild-type male mosquitoes could be released at numbers comparable to those described here (or possibly lower, especially if wild-type mosquitoes have a fitness advantage) in order to eliminate the transgene. Alternatively, should pathogens evolve resistance to the vector's expressed anti-pathogen mechanisms or to become more virulent to humans (e.g., [65]), we anticipate that releases using alternative or complementary transgenic constructs ([27]) may be considered even without the need to successfully link all such constructs to a gene-drive mechanism. However, releases with multiple constructs may also raise the effective fitness costs experienced by transgenic strains substantially.

Following previous modeling studies (e.g., [27], [41] and [39]), we considered an additive fitness cost of 5–10% per copy of the anti-pathogen gene. We found that although such a fitness cost could reduce the long-term frequency of vector-incompetent females, the rate at which the vector-competent population recovered was relatively mild (e.g., Fig. 1) especially when compared to the rate of recovery of the vector-competent population in a RR or FK-only strategy (e.g., Fig. 4). Nevertheless, some lab- and field cage-based studies have reported that fitness costs associated with transgenic insertions in *Ae. aegypti* can potentially be much larger or operate at different life stages (e.g., [66] and [67]). How such fitness costs are expressed under field conditions remains an open question (e.g., [68]), although improvements have been made in reducing fitness costs at least in artificial settings ([69]). As specific anti-pathogen constructs become available, our comparative framework provides one approach to assessing the release numbers necessary to compensate for different reported fitness costs. Such comparative assessments could be used to discern if the release numbers required are prohibitive, and can provide quantitative evidence for the need to invest in developing gene-drive like technologies, further reducing the fitness cost, or alternative anti-pathogen transgenes. Finally, in some cases the dengue virus may confer a fitness cost on infected mosquitoes (e.g., [43]), providing a potential fitness benefit to transgenic mosquitoes that can facilitate spread without being linked to a gene-drive mechanism ([29]). Our results indicate that even without conferring a fitness benefit, an anti-pathogen transgene may substantially reduce vector competence in an urban *Ae. aegypti* population. However, if carrying a transgene provides a net fitness benefit to mosquitoes in the field, then under certain conditions (e.g., if there is sufficient assortative mating among transgenic mosquitoes - e.g., [30]), releasing more mosquitoes might not be necessary to compensate for fitness costs associated with the transgene. In light of our results that population replacement may not require gene-drive, assessing any potential fitness benefits transgenic constructs provide their bearers, as well as quantifying the refractoriness provided by the anti-pathogen construct, become particularly salient issues.

Some transgenic control programs, particularly those based on population reduction, frequently apply a density-independent control method or wait for seasonally low densities before conducting releases ([70]). For instance, [51] simulated a two-week, pre-release vector control program using a traditional method (e.g., insecticide spraying) that lowered the number of released mosquitoes carrying a dominant, conditionally lethal-construct necessary to achieve extinction. Some preliminary analyses show, in principle, that a similar approach could potentially improve prospects for population replacement, although the duration of the traditional intervention needs to be somewhat longer to have a detectable effect when release sizes are small (Figure S6). A possible further advantage of having population reduction precede transgenic population replacement efforts (rather than aim to simultaneously reduce and replace a vector population) is that it could decouple an anti-pathogen construct from the conditionally-lethal gene. This could mitigate any detrimental effects transient linkage disequilibria may have when the two constructs are released simultaneously (as may occur in the RR strategy - [32]). Using a pre-release control that can rapidly cause significant population reductions (e.g., spraying) may therefore help raise the effective release ratio and provide an alternative to implementing a genetic replacement strategy over an extended period of time or, where rearing or fitness costs are potentially high, allow even fewer mosquitoes to be released than the numbers considered here.

In conclusion, our results raise an intriguing possibility: even in the absence of drive mechanisms or fitness advantages conferred by transgenic constructs, releasing mosquitoes in numbers much smaller than those considered necessary for other genetic management strategies (e.g., those based on population reduction) can result in rapid and robust population replacement. Although smaller release numbers may suffice to establish *Wolbachia* in some settings (e.g., [71]), alternative, engineered anti-pathogen genes may continue to be necessary in responding to possible failures of *Wolbachia* (or any other specific anti-dengue construct) to reduce disease transmission in the field. Directing efforts to improve rearing capacity and logistical support for implementing releases, and reducing the fitness costs of existing recombinant technologies, may provide a viable, alternative route to introgressing anti-pathogen transgenes under field conditions.

## Supporting Information

Figure S1 The average reduction in vector-competent females from 30 simulation runs two years after releases end when both male and female transgenic mosquitoes are released (A) at all sites for a single year, (B) at all sites for three years, (C) at 10% of the sites across a regular grid for a single year, and (D) at 10% of the sites across a regular grid for three years. In panels (C) and (D), males and females are released in equal numbers. In all panels, the black lines represent results in the presence of a fitness cost associated with the anti-pathogen transgene, and grey lines represent the results assuming no fitness cost. The end points on the error bars represent the 2.5th and 97.5th percentile abundances across simulation runs.
(EPS)

Figure S2 A comparison of the reduction in competent female vectors across three transgenic control strategies: direct population reduction (an FK strategy aiming exclusively at population reduction), a “reduce and replace” (an RR strategy) strategy, and the “replacement-alone” strategy (an AP strategy), when adult male transgenic mosquitoes are released in all sites, which is the scenario that provided the highest extinction rates for transgenic control measures based on vector population reduction alone (e.g., [51] - we note that female releases are assumed to be undesirable when released females carry only a conditionally-lethal gene). In both panels, the lines represent the number of vector competent females averaged over all 30 model runs, and thus incorporate simulations resulting in population extinction. Panel (A) describes the results of modeling the release of 4 males per site for a single and three years, while panel (B) describes the results of modeling the release of 8 males per site for a single year. In both panels, grey lines represent an AP strategy, black lines represent an RR strategy, and blue lines represent a strategy based on population reduction alone. For the RR and AP strategies (black and grey lines), solid lines represent scenarios with a fitness cost associated with the anti-pathogen transgene, while dashed lines represent scenarios where no fitness cost is associated with the anti-pathogen transgene. For the strategy based on population reduction alone, the solid line represents a single year release while the dashed line represents a three year release. Here, and in Supporting Information Figure S3, we assume that the conditionally-lethal construct carries no additional fitness costs beyond dominant female adult lethality.
(EPS)

Figure S3 The average number of vector competent mosquitoes for each day across 30 simulation runs modeling “reduce and replace” (RR) transgenic control strategies and a strategy releasing mosquitoes carrying the anti-pathogen construct alone (an AP strategy). The same release regimes are analyzed as in Figure 4 in the main text, but unlike Figure 4 in the main text, simulations resulting in population elimination are not omitted in calculating the daily average number of vector competent mosquitoes. (A) adult males are released at all sites, (B) adult male and adult female releases at all sites, (C) eggs are released in 10% of the sites along a regular grid and (D) adult males and females are released in 10% of the sites along a regular grid. In all panels, solid lines represent releases for a single year and dashed lines represent 3 years releases. RR releases are illustrated in black and AP only releases are in grey. The numbers of mosquitoes released per site for the illustrated time series are: (A) 2 adult males, (B) 1 adult male and 1 adult female, (C) 100 eggs, (D) 10 adult males and 10 adult females.
(EPS)

Figure S4 The average number of vector competent mosquitoes for each day across 30 simulation runs modeling “reduce and replace” (RR) transgenic control strategies and a strategy releasing mosquitoes carrying the anti-pathogen construct alone (an AP strategy) under differing fitness costs associated with the anti-pathogen gene. The same release regimes are analyzed as in Figure 5 in the main text, but unlike Figure 5 in the main text, simulations resulting in population elimination are not omitted in calculating the daily average number of vector competent mosquitoes. The results are for (A) adult male releases at all sites, (B) adult male and adult female releases at all sites, (C) adult male releases at 10% of the sites along a regular grid, and (D) adult male and adult female releases at 10% of the sites along a regular grid. In all panels, dotted lines represent the absence of a fitness cost associated with the anti-pathogen gene, solid lines represent a fitness cost of 5% per copy of the anti-pathogen gene, and dashed lines represent a fitness cost twice the value used in previous figures (10% fitness cost per copy). RR releases are illustrated in black and AP-only releases are in grey. We illustrate the time series from a single year of releases with per-site release numbers of (A) 7 adult males, (B) 1 adult male and 1 adult female, and (C) 80 adult males, and (D) 15 adult males and 15 adult females.
(EPS)

Figure S5 An illustration of minimal spatial variation in the dynamics of transgenic and wild-type adult females through space and time for a single run. Results are from a simulated weekly release of 6 males at all sites for one year. The anti-pathogen transgene is assumed to carry a fitness cost of 5% per copy. Colors represent the frequency of female adults carrying the anti-pathogen gene at each site, from blue (wild-type only) through red (anti-pathogen gene is at fixation). The number at the top of each panel represents the frequency of adult females carrying the anti-pathogen gene on the corresponding date.
(EPS)

Figure S6 A comparison of the average reduction across 10 simulation runs in competent female vectors with and without pre-release traditional control interventions (e.g., chemical insecticide spraying). The dashed line represents the reduction in the absence of a pre-release traditional control intervention, the dotted line represents the reduction with a pre-release traditional control intervention lasting two weeks, and the solid line represents the reduction assuming a pre-release traditional control intervention lasting two months before releases begin. The simulations model the release of 100 eggs in 10% of the sites randomly determined for a single year. In all control situations, the anti-pathogen transgene is assumed to carry a fitness cost of 5% per copy.
(EPS)

Text S1 A description of how the release area for the *Wolbachia*-based field trials were measured.
(PDF)
