# Supplementary material for: Feasible Introgression of an Anti-pathogen Transgene into an Urban Mosquito Population without Using Gene-Drive
Source: PLoS Negl Trop Dis. 2014 Jul 3;8(7):e2827. doi: 10.1371/journal.pntd.0002827 (PMC4081001; doi:10.1371/journal.pntd.0002827)
Supplement: Text S1 — A description of how the release area for the Wolbachia-based field trials were measured. (PDF) [file pntd.0002827.s007.pdf]

Supplementary Information S1 Measuring the release area for the *Wolbachia*-based field trials

Because [1] do not specifically report the size of the areas where releases occurred, we estimated the size of the release area based on the regions illustrated in Supplementary Fig. S2 of [1]. We manually traced the perimeter of the release regions specified in [1] and used an automated area calculator ([2]) based on Google Maps ([3]) to estimate the size of the release area enclosed within the perimeter. Based on this analysis, we found the total release area to encompass a region of approximately 200 hectares.

References

1. Hoffmann AA, Montgomery BL, Popovici J, Iturbe-Ormaetxe I, Johnson PH, et al. (2011) Successful establishment of *Wolbachia* in *Aedes* populations to suppress dengue transmission. Nature 476: 454–7.

2. ACME (2013). Google Planimeter. URL `http://acme.com/planimeter/`.

3. Google Incorporated (2013). Google Maps. URL `maps.google.com`.
